# Variation in regional implantation patterns of cardiac implantable electronic device in Switzerland

**DOI:** 10.1371/journal.pone.0262959

**Published:** 2022-02-16

**Authors:** Lucy Bolt, Maria M. Wertli, Alan G. Haynes, Nicolas Rodondi, Arnaud Chiolero, Radoslaw Panczak, Drahomir Aujesky

**Affiliations:** 1 Department of General Internal Medicine, Inselspital, Bern University Hospital, University of Bern, Bern, Switzerland; 2 CTU Bern, University of Bern, Bern, Switzerland; 3 Institute of Primary Health Care (BIHAM), University of Bern, Bern, Switzerland; 4 Population Health Laboratory (#PopHealthLab), University of Fribourg, Fribourg, Switzerland; 5 School of Population and Global Health, McGill University, Montreal, Canada; 6 Institute of Social and Preventive Medicine, University of Bern, Bern, Switzerland; Karolinska Institutet, SWEDEN

## Abstract

**Introduction:**

There is a substantial geographical variation in the rates of pacemaker (PM), implantable cardioverter defibrillator (ICD), and cardiac resynchronization therapy (CRT) device implantation across European countries. We assessed the extent of regional variation and potential determinants of such variation.

**Methods:**

We conducted a population-based analysis using discharge data for PM/ICD/CRT implantations from all Swiss acute care hospitals during 2013–2016. We derived hospital service areas (HSA) by analyzing patient flows. We calculated age- and sex-standardized rates and quantified variation using the extremal quotient (EQ) and the systemic component of variation (SCV). We estimated the reduction in variance of crude implantation rates across HSAs using multilevel regression models, with incremental adjustment for age and sex, language, socioeconomic factors, population health, diabetes mellitus, and the density of cardiologists on the HSA level.

**Results:**

We analyzed implantations of 8129 PM, 1461 ICD, and 1411 CRT from 25 Swiss HSAs. The mean age- and sex-standardized implantation rate was 29 (range 8–57) per 100,000 persons for PM, 5 (1–9) for ICD, and 5 (2–8) for CRT. There was a very high variation in PM (EQ 7.0; SCV 12.6) and ICD (EQ 7.2; SCV 11.3) and a high variation in CRT implantation rates (EQ 3.9; SCV 7.1) across HSAs. Adjustments for age and sex, language, socioeconomic factors, population health, diabetes mellitus, and density of cardiologists explained 94% of the variance in ICD and 87.5% of the variance in CRT implantation rates, but only 36.3% of the variance in PM implantation rates. Women had substantially lower PM/ICD/CRT implantation rates than men.

**Conclusion:**

Switzerland has a very high regional variation in PM/ICD implantation and a high variation in CRT implantation rates. Women had substantially lower implantation rates than men. A large share of the variation in PM procedure rates remained unexplained which might reflect variations in physicians’ preferences and practices.

## Introduction

Pacemakers (PM), implantable cardioverter defibrillators (ICD), and cardiac resynchronization therapy (CRT) devices are commonly used cardiac implantable electronic devices (CIED). PMs have the potential to improve quality of life in symptomatic patients with sinus node dysfunction or atrioventricular block and show a survival benefit in patients with infranodal atrioventricular block [1, 2]. Further, ICDs are used to prevent sudden cardiac death (SCD) in patients who are at an increased risk or have sustained ventricular tachycardia or fibrillation [3–5]. CRTs improve cardiac function in selected patients with severe heart failure [2, 4, 5].

Before implanting a CIED, its potential benefit needs to be carefully weighed against the periprocedural risk and potential adverse effects. Periprocedural complications such as lead dislodgement, hematoma, pneumothorax, and infections occur in approximately 3–20% of PM [1, 2, 6] and in approximately 10% of ICD [6] and 11% of CRT device implantations [2, 6]. Further, inappropriate shocks of ICD devices have been observed in approximately 20% of patients [5, 7].

**Fig 1:**
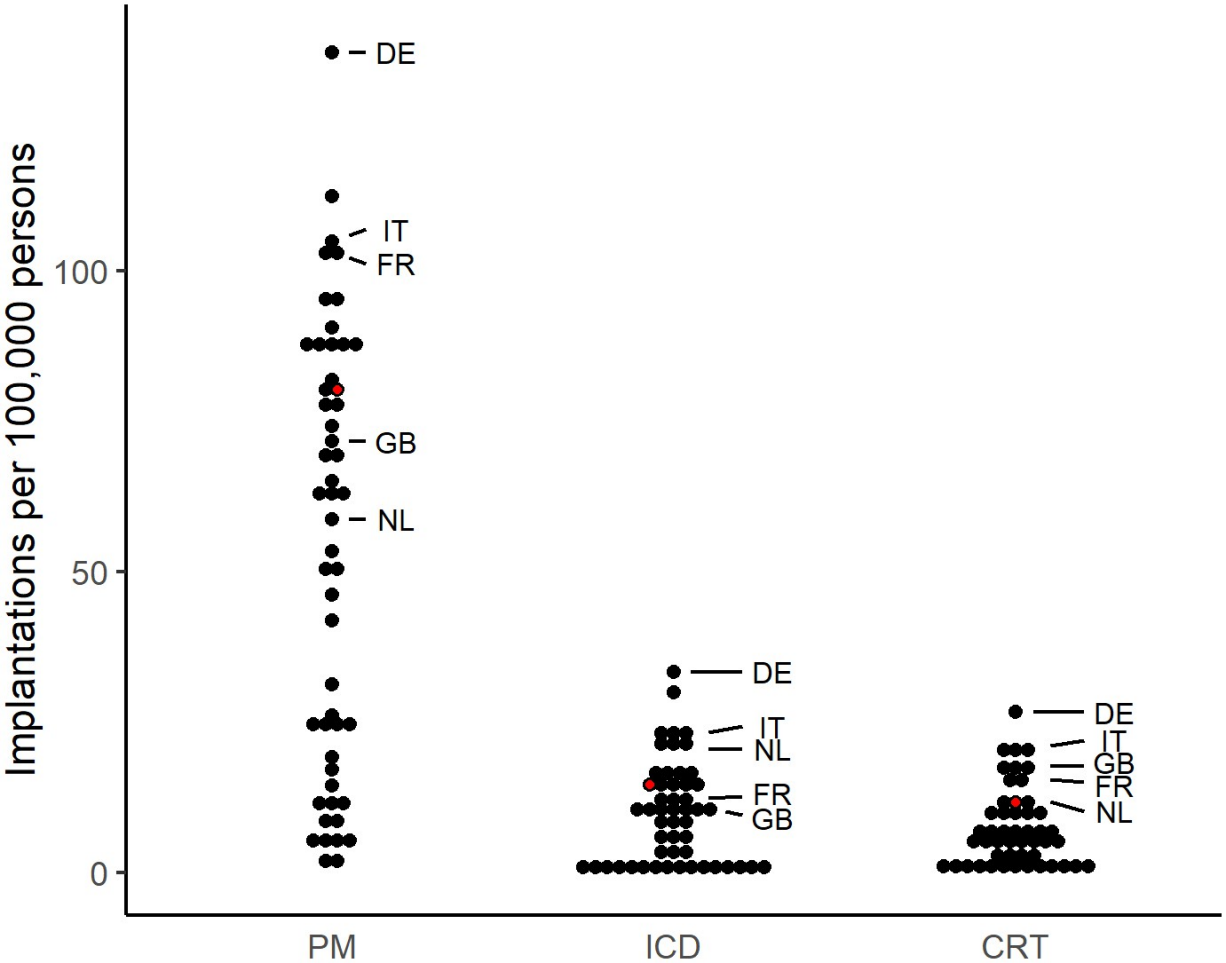
Variation in average CIED implantation rates per 100,000 persons across member countries of the European Society of Cardiology in 2016. Switzerland is represented as a red dot. Data extracted from the European Heart Rhythm Association [8]. Abbreviations: CIED = cardiac implantable electronic device, PM = pacemaker, ICD = implantable cardioverter defibrillator, CRT = cardiac resynchronization therapy, DE = Germany, IT = Italy, FR = France, GB = United Kingdom, NL = Netherlands.

The average CIED implantation rate increased substantially between 2007 and 2016 (PM by 20%, ICD by 44%, and CRT by 121%) in the 56 member countries of the European Society of Cardiology (ESC), with a substantial between-country variation (Fig 1) [8]. Although variation in CIED implantation rates has also been reported within countries [9–12], a systematic review concluded that publications were heterogeneous with regard to the data source, time period, geographic context, type of examined device, and the detail level of the analysis [13]. Whereas in some countries all CIED implantations are included in a national registry (e.g., Denmark, and Sweden), other national registries only include voluntary data from specialists (e.g., Portugal and Spain) or survey data [14], which may introduce variation or limit representativeness [15, 16]. Most previous studies used data of CIED implantations up until 2012 only that may no longer be accurate. More recently published studies also used registry data (e.g., [16–19]) or included older data (e.g., [15, 20, 21]). Geographical variations in care are associated with a multitude of factors, including demographic, socioeconomic, and health system related factors (e.g., supply, access, reimbursement), and patient and physician preferences [22–28]. However, only few studies used statistical modelling to explore the influence of potential determinants on such variation [13].

Compared to its neighboring countries, Switzerland had the highest growth rate for PM (+21%) and the second highest for CRT devices (+114%) between 2007 and 2016 [8]. Switzerland has universal health care coverage, good access to care, the same nationwide reimbursement system, and a very low regional variation in cardiovascular mortality [29], indicating similar cardiovascular disease incidences across Swiss regions. Furthermore, the close proximity of a population with different socioeconomic and cultural factors is ideal to explore potential differences that influence treatment decisions. We hypothesized that the regional variation in CIED procedures would be low in Switzerland. Our aim was to assess the variation in CIED device implantation rates across Swiss regions and to assess which regional demographic, socioeconomic, health, and supply-related factors explain such variation.

## Methods

### Data sources

We conducted a population-based, small area variation analysis based on routinely collected patient discharge data from all public and private Swiss acute care hospitals and Swiss census data for calendar years 2013–2016. The methods have been described previously [30, 31]. In summary, Swiss hospitals are legally obligated to provide the Swiss Federal Statistical Office (SFSO) with an anonymized, standardized data set for each hospital discharge. These data are centrally stored in the Swiss Hospital Discharge Masterfile hosted at the SFSO. Recorded variables include patient age, sex, nationality, insurance type, up to 100 procedure codes based on the Swiss Classification of Operations (CHOP, an adaptation of the U.S. ICD-9-CM volume 3 procedure classification), up to 40 diagnostic codes based on the International Classification of Diseases, and 10^th^ revision, German Modification (ICD-10-GM). Further, the area of patient residence and hospital location within one of 705 Swiss administrative regions (MedStat regions) based on aggregated ZIP-codes is recorded [32]. The SFSO reviews data integrity and completeness by means of a specifically designed software [33]. As hospital reimbursement is directly based on the documentation of the main procedures, data completeness and accuracy of CHOP codes is likely to be very high [34].

We used Swiss National Cohort data [35] and community typology data [36] from 2014 to determine the language (Swiss German vs. French/Italian) and population data to determine population density for each MedStat region. We used the mean Swiss Socioeconomic Position (SSEP, version 2) as a measure of socioeconomic status [37]. The SSEP version 2 was derived using 2012–2015 Swiss structural survey data to rank Swiss neighborhoods based on four domains (median rent/m^2^, proportion of households led by a person with no/low education, proportion headed by a person in manual/unskilled occupation, and mean crowding, all on the neighborhood level). The SSEP varies between zero (lowest) and 100 (highest) and correlates well with mortality [37]. Finally, we obtained the density of cardiologists per MedStat region for calendar year 2014 from the Swiss Medical Association (FMH). Our study was based on anonymized administrative data only and was thus exempted from ethics committee approval according to the Swiss Human Research Act.

### Derivation of hospital service areas (HSA)

Switzerland has compulsory basic health insurance coverage, with voluntary semiprivate and private insurance plans covering additional medical services. Although Swiss hospital care is primarily organized based on 26 administrative regions (the cantons), patients may utilize hospital services outside their canton of residence and the use of cantons as a unit of observation may skew procedure rates. We therefore used a fully automated method to generate reproducible, general hospital service areas (HSAs) using all patient discharge data from the calendar years 2013–2016 [30]. Only patients living in Switzerland were considered. In a first step, we identified 4,105,885 patient discharges aged ≥18 years from 155 Swiss acute care hospitals during calendar years 2013–2016 (S1 Fig). We then identified MedStat regions with ≥1 hospital and analyzed patient flows from neighboring MedStat regions. MedStat regions that had the highest proportion of their residents discharged from the same hospital MedStat region were assigned to the same HSA (plurality rule). HSAs with <50% of the patients being treated within the same HSA or <10 discharges overall were merged with the neighboring HSA which received most discharges until >50% and ≥ 10 discharges occurred within each HSA. This process yielded 63 general HSAs. In a second step, we identified patient discharges with specific CHOP codes for a first-time PM implantation (CHOP codes 37.80, 37.81.00/10, 37.81.99, 37.82, 37.83, 37.88, 37.8A.00/11/21-22/4), first-time ICD implantation (CHOP codes 37.8E.00, 37.8E.1, 37.8E.22, 37.8E.3, 37.94.00–05, 37.99), and first-time CRT implantation (CRT with pacemaker function CHOP codes 00.50 and 37.8A.3; CRT with an incorporated defibrillator 00.51 and 37.8E.21). As CIED implantations are not performed in all Swiss hospitals, we analyzed patient flows for all CIED procedures using the same method described above, further aggregating the 63 general HSAs in 25 procedure-specific HSAs in which all 3 procedures were performed. We then drew choropleth maps of the 25 final HSAs using Geographical Information System (GIS)-compatible vector files.

### Study population

Overall, we identified 23,635 adult discharges with specific codes for CIED device implantations (S1 Fig). We excluded all discharges related to emergency procedures (n = 7707), self-harm (ICD 10 codes X60—X84, n = 122), temporary PM (n = 1832), and CIED removal during the same hospitalization as the implantation (n = 2973), leaving a final study sample of 8129 patient discharges for PM implantation, 1461 for ICD implantation, and 1411 for CRT implantation.

### Measures of variation

We calculated age- and sex-standardized CIED procedure rates per 100,000 persons for each HSA using procedure counts and 2013–2016 census data for the adult Swiss population [38]. We used direct standardization using age bands of 18 to 49, 50 to 59, 60 to 69, 70 to 79, and ≥80 years. To examine the variation in procedure rates across Swiss HSAs, we determined the extremal quotient (EQ), which is the highest divided by the lowest procedure rate. While the EQ is an intuitive measure of variation, it is prone to distortion by extreme values [39]. We also calculated the coefficient of variation (CV), i.e., the ratio of the standard deviation of the procedure rates to the mean rate, and the systematic component of variation (SCV) [39, 40]. Although less intuitive than the EQ, the SCV represents the non-random part of the variation in procedure rates while reducing the effect of extreme values [39–41]. The SCV is derived from a model that recognizes the differences in rates across areas and the random variation within each area’s true rate. A SCV of 5.4–10 is considered indicative of a high variation and a SCV of >10 of a very high variation [39, 41].

### Determinants of variation

We explored whether the following HSA-level factors influenced CIED procedure rates: demographics (age and sex strata), language region (Swiss German vs. French/Italian), socioeconomic factors (population density, socioeconomic status, insurance class, Swiss citizenship), population health, and supply factors (density of cardiologists). We used population density as a proxy for neighborhood residence (e.g., rural areas have a low population density, cities generally have a higher density). The socioeconomic status of each HSA was measured using the mean value of the SSEP of persons within an HSA. As only wealthier persons can buy additional health insurance [42] and Swiss nationals tend to be wealthier [43] and have a better education than foreign nationals residing in Switzerland [43] we used the percentage of all hospital discharges with (semi-)private insurance status and Swiss citizenship as an additional measure of socioeconomic status for each HSA. As a proxy for the population health, we calculated age-standardized incidence rates of hip fractures (ICD 10 codes S72.0—.2), colon (ICD 10 codes C18/19 and CHOP codes 45.7- .8) or lung cancer (ICD 10 codes C34 and CHOP codes 32.3 -.6 or 32.9) treated surgically, acute myocardial infarctions (ICD 10 codes I21), or strokes (ICD 10 codes I63/64) for each HSA, as differences in these disease rates are likely to reflect true regional differences in population health rather than differences in coding intensity or supply factors [44, 45]. Because diabetes mellitus is a known risk factor for cardiovascular disease [46], we used the proportion of hospitalized patients with diabetes in each HSA as a further measure of population health. The density of cardiologists per HSA was used as a supply measure.

### Statistical analyses

We used progressively adjusted multilevel Poisson regression with a log link to model the procedure rates in each HSA using age bands of 18–49, 50–59, 60–69, 70–79 and ≥80 years. HSA was included as a random intercept in all models. In a progressive approach, model 1 was adjusted for the calendar year of the procedure, model 2 in addition for demographics, model 3 added language region and socioeconomic factors, model 4 population health, and model 5 supply factors. Variables included in the model were chosen *a priori* as we expected them to influence the rates. Due to the high correlation (r = 0.81) between the SSEP and (semi-)private insurance status, we only included the insurance status as a proxy of the socioeconomic wealth of an area to reduce variance inflation and collinearity. The models were used 1) to quantify the effect of explanatory factors on CIED implantation rates, 2) to assess the variance explained by the factors defined previously, and 3) to calculate adjusted CIED rates per 100,000 persons per HSA.

We expressed the effect of explanatory factors on CIED implantation rates as incidence rate ratios (IRRs), defined as the CIED implantation rate in the defined category (e.g., French/Italian language region) relative to the estimated CIED implantation rate in the reference category (e.g., Swiss German language region). The reduction in variation across the HSAs was determined by examining the variance of the random intercept relative to model 1. We considered the residual, unexplained variation of the fully adjusted model a proxy for unwarranted variation. Finally, we used the fully adjusted models to predict CIED implantation rates in each HSA. Separate sets of models were created for PM, ICD, and CRT devices. Statistical modeling was performed using Stata version 15.1 (StataCorp, College Station, TX, USA). HSAs were delineated and maps drawn using the R statistical software, version 3.4.2 [47].

## Results

### Characteristics of Swiss HSAs and the study population

The median population size per HSA was 165,172 persons (interquartile range [IQR] 88,524–363,164), with a median population density of 307 persons per km^2^ (IQR 199–392), and a median cardiologist density of 6.8 per 10,000 persons (IQR 5.3–8.2). Seventeen HSAs were located in Swiss German-speaking and 8 in French-/Italian-speaking regions.

Overall, 79.7% of patients with PM, 27.6% with ICD, and 56.6% with CRT implantation were aged 70 years or older (Table 1). The majority of patients who underwent CIED implantation were men (PM 59.6%, ICD 81.2%, CRT 73.9%), had a general insurance coverage (PM 60.6%, ICD 76.6%, CRT 66.3%), and were Swiss nationals (PM 90.5%, ICD 80.4%, CRT 85.1%).

**Table 1:** Characteristics of the study population with CIED implantations in Switzerland from 2013 to 2016.

	PM (N = 8129)	ICD (N = 1461)	CRT (N = 1411)
	n (%)		
Case year			
2013	1,873 (23.0)	318 (21.8)	288 (20.4)
2014	2,001 (24.6)	346 (23.7)	329 (23.3)
2015	2,106 (25.9)	398 (27.2)	399 (28.3)
2016	2,149 (26.4)	399 (27.3)	395 (28.0)
Age			
18–49	202 (2.5)	254 (17.4)	60 (4.3)
50–59	316 (3.9)	309 (21.1)	166 (11.8)
60–69	1,139 (14)	494 (33.8)	386 (27.4)
70–79	2,964 (36.5)	354 (24.2)	562 (39.8)
≥80	3,508 (43.2)	50 (3.4)	237 (16.8)
Male sex	4,844 (59.6)	1,187 (81.2)	1,042 (73.9)
Insurance class			
General	4,930 (60.6)	1,119 (76.6)	936 (66.3)
(Semi-)private	3199 (39.3)	342 (23.4)	475 (33.6)
Swiss citizenship	7,354 (90.5)	1,174 (80.4)	1,201 (85.1)

Abbreviations: CIED = cardiac implantable electronic device, PM = pacemaker, ICD = implantable cardioverter defibrillator, CRT = cardiac resynchronization therapy.

### Variation in procedure rates across HSAs

**Fig 2:**
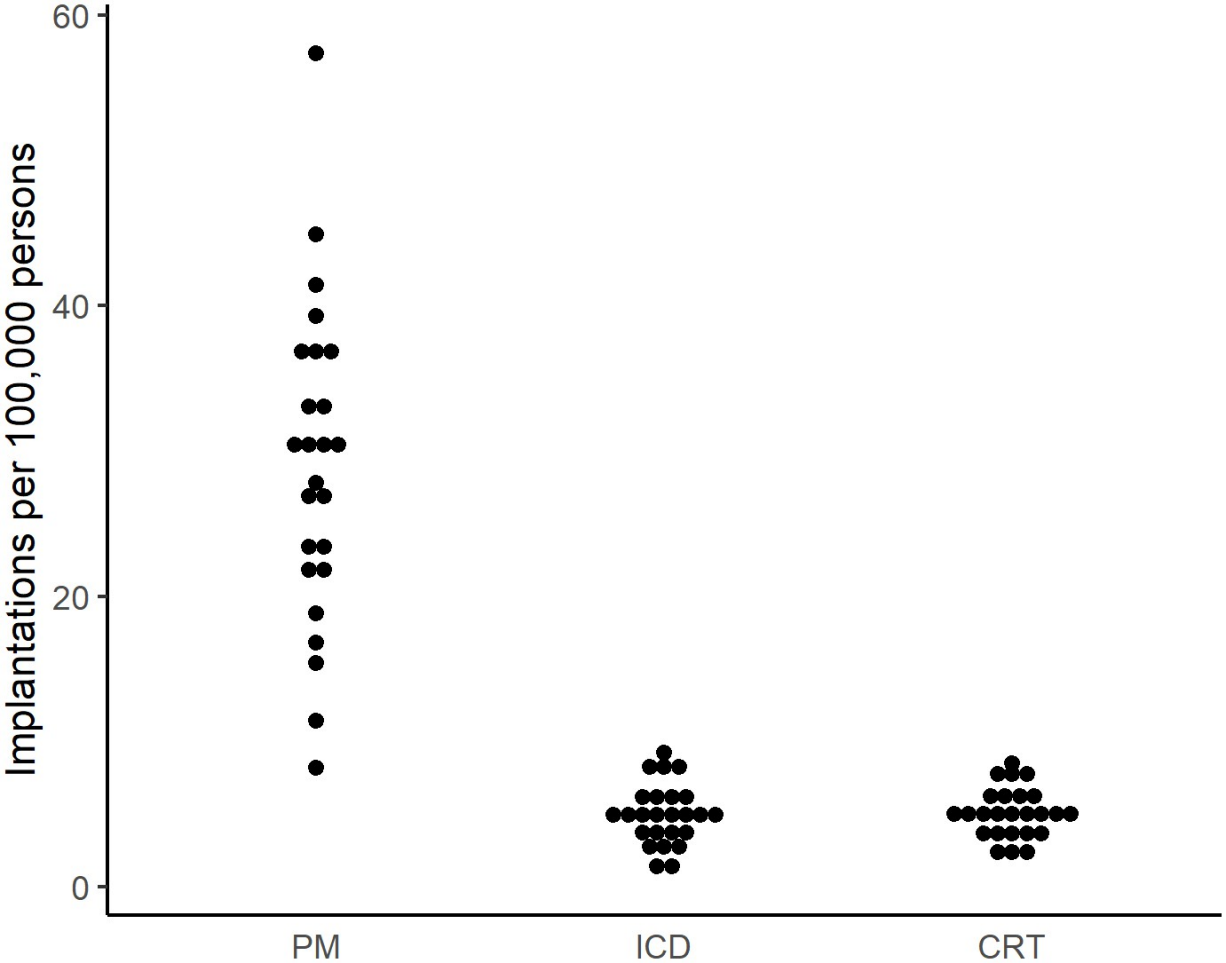
Variation in age- and sex-standardized CIED implantation rates per 100,000 persons across 25 Swiss hospital service areas between 2013 and 2016. Abbreviations: CIED = cardiac implantable electronic device, PM = pacemaker, ICD = implantable cardioverter defibrillator, CRT = cardiac resynchronization therapy.

The mean age- and sex-standardized implantation rate for PM, ICD, and CRT was 29 (range 8–57), 5 (1–9), and 5 (2–8) per 100,000 persons, respectively. The variation in implantation rates across HSAs was very high for PMs (EQ 7.0, CV 0.4, SCV 12.6) and ICD devices (EQ 7.2, CV 0.4, SCV 11.3), and high for CRT (EQ 3.9, CV 0.3, SCV 7.1). Fig 2 depicts the variation in mean age- and sex standardized PM, ICD, and CRT device implantation rates across HSAs. Detailed age- and sex-standardized PM, ICD and CRT procedure rates for each HSA are shown in the S1 Table.

**Fig 3:**
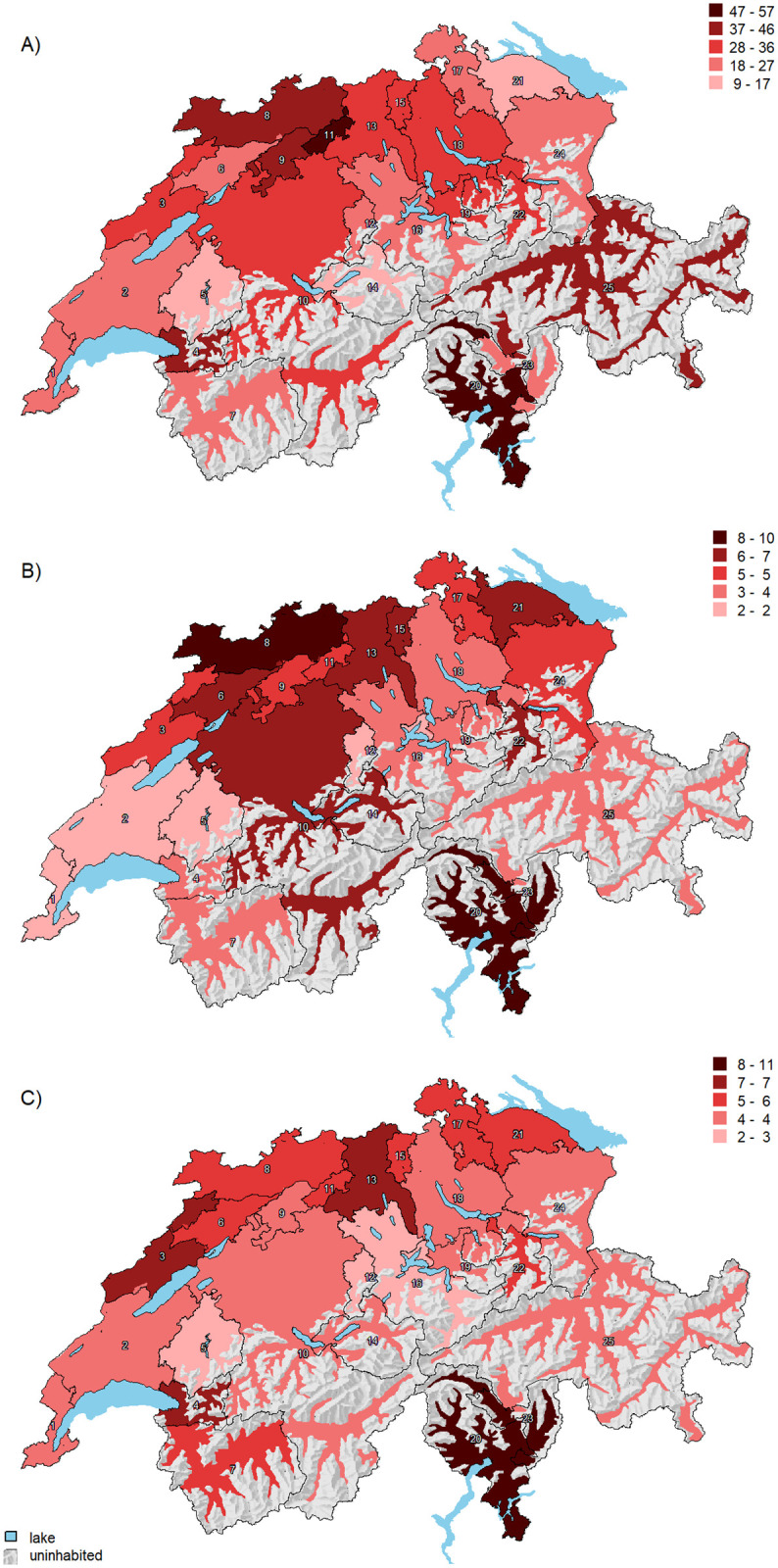
Adjusted average predicted CIED implantation rates across 25 Swiss hospital service areas. Panel A: PM, Panel B: ICD, Panel C: CRT. Average predicted procedures rates for each HSA are shown as red-scale categories per 100,000 persons. Adjusted for year, age, sex, language region, population density, insurance status, Swiss citizenship, population health, diabetes mellitus, and the density of cardiologists. Abbreviations: CIED = cardiac implantable electronic device, PM = pacemaker, ICD = implantable cardioverter defibrillator, CRT = cardiac resynchronization therapy, HSA = hospital service area. Shaded relief map reprinted from the Federal Office of Topography swisstopo, Switzerland https://shop.swisstopo.admin.ch/en/products/maps/overview/relief and shape files derived from postcode-level shape file used to create map of Switzerland, e.g., https://www.geocat.admin.ch/) under a CC BY license, with permission from Alexandra Frank, original copyright 2006.

After full adjustment for demographics, language, socioeconomic factors, population health, and the density of cardiologists, the predicted PM implantation rates varied between 10.2 and 55.5 per 100,000 persons across HSAs (Fig 3, Panel A). Two HSAs had PM implantation rates above 46 per 100,000 persons (HSA number 11, and 20) and 3 below 18 per 100,000 persons (HSA number 5, 14, and 21). The predicted ICD implantation rates varied between 2.1 and 9.2 per 100,000 persons (Fig 3, Panel B). Three HSAs had an implantation rate above 8 per 100,000 persons (number 8, 20, and 23) and four below 3 per 100,000 persons (number 1, 2, 5, and 12). For CRT, the predicted CRT rates varied between 2.5 and 9.6 per 100,000 persons across HSAs. Two HSAs had an implantation rate above 7 per 100,000 persons (Fig 3, Panel C, HSA number 20 and 23) and three HSAs below 4 per 100,000 persons (number 5, 12, and 16).

### Determinants of variation in implantation rates

Procedure year was not associated with procedure rates (Table 2). While PM implantation rates continued to increase significantly with increasing age (IRR 7.27, 95% confidence interval [CI] 6.80–7.77 for patients aged ≥80 years compared to patients aged 60–69 years), ICD implantation rates peaked at 60–79 years and CRT rates at 70–79 years of age. Men had significantly higher CIED implantation rates than women. None of the other examined predictors was associated with PM implantation. Compared to Swiss German speaking areas, French-/Italian speaking areas had lower (IRR 0.66, 95% CI 0.50–0.86), whereas areas with a lower population health (IRR 3.37, 95% CI 1.67–6.82 per comorbidity per 1000 persons) and a higher prevalence of diabetes mellitus (IRR 3.69, 95% CI 1.90–7.16 per 10% change in prevalence) had higher ICD implantation rates. Similarly, areas with a higher proportion of persons with Swiss citizenship had a lower (IRR 0.73, 95% CI 0.57–0.93 per 10%) and those with a higher prevalence of diabetes mellitus (IRR 2.08, 95% CI 1.06–4.09 per 10% change in prevalence) had higher CRT implantation rates. Neither population nor cardiologist density were statistically significantly associated with procedure rates.

**Table 2:** Determinants of CIED implantation rates across 25 Swiss hospital service areas.

		PM	ICD	CRT
		IRR (95%-CI)*		
Case year	2013	Reference	Reference	Reference
	2014	1.01 (0.94–1.08)	0.95 (0.81–1.11)	1.03 (0.88–1.21)
	2015	1.00 (0.92–1.10)	0.94 (0.80–1.10)	1.14 (0.96–1.34)
	2016	0.98 (0.88–1.09)	0.85 (0.72–1.02)	1.05 (0.88–1.25)
Age	18–49	**0.04 (0.04–0.05)**	**0.13 (0.11–0.15)**	**0.04 (0.03–0.05)**
	50–59	**0.21 (0.18–0.23)**	**0.46 (0.40–0.53)**	**0.32 (0.26–0.38)**
	60–69	Reference	Reference	Reference
	70–79	**3.73 (3.49–4.00)**	1.05 (0.91–1.20)	**2.11 (1.86–2.41)**
	≥80	**7.27 (6.80–7.77)**	**0.26 (0.20–0.35)**	**1.54 (1.31–1.81)**
Sex	Men	Reference	Reference	Reference
	Women	**0.50 (0.48–0.52)**	**0.22 (0.19–0.25)**	**0.30 (0.26–0.34)**
Language region	Swiss German	Reference	Reference	Reference
	French/Italian	0.87 (0.59–1.28)	**0.66 (0.50–0.86)**	0.84 (0.64–1.10)
Population density (per 100 persons change per km^2^)		1.02 (0.98–1.07)	1.02 (0.99–1.06)	1.02 (0.99–1.05)
(Semi)private insurance (per 10% change)		1.01 (0.75–1.36)	0.83 (0.67–1.04)	0.81 (0.65–1.01)
Swiss citizenship (per 10% change)		0.84 (0.65–1.09)	0.89 (0.70–1.13)	**0.73 (0.57–0.93)**
Population health (per comorbidity/1000 persons)†		1.07 (0.68–1.69)	**3.37 (1.67–6.82)**	1.69 (0.78–3.68)
Diabetes mellitus (per 10% change in prevalence)		1.63 (0.93–2.89)	**3.69 (1.90–7.16)**	**2.08 (1.06–4.09)**
Density of cardiologists (per 1 cardiologist/10,000 persons)		0.94 (0.87–1.02)	1.01 (0.96–1.06)	0.98 (0.93–1.03)

Abbreviations: CIED = cardiac implantable electronic device, PM = pacemaker, ICD = implantable cardioverter defibrillator, CRT = cardiac resynchronization therapy, IRR = incidence rate ratio, CI = confidence interval.

*Procedure rate in the defined category relative to the procedure rate in the reference category. For instance, an IRR of 0.50 indicates a 50% lower PM implantation rate in women than in men.

^†^Population health is defined as the sum of age-standardized incidence rates for the following comorbidities: hip fracture, colon or lung cancer treated surgically, acute myocardial infarction, and stroke.

Table 3 shows the reduction in variance of CIED implantation rates with incremental adjustment for demographics, language, socioeconomic factors, population health, and density of cardiologists. Adjustment of language/socioeconomic factors (13.7% for PM, 34.6% for ICD, and 47.4% for CRT) and population health and diabetes mellitus (47.3% for ICD and 30.1% for CRT) resulted in the greatest reduction in the variance of implantation rates. The fully adjusted model explained 36.3% of the variance of PM, 94.0% of ICD, and 87.5% of CRT implantation rates.

**Table 3:** Remaining variance after incremental adjustment for potential determinants of CIED implantations across 25 Swiss hospital service areas.

	Model 2†	Model 3‡	Model 4§	Model 5¶
	% of variance			
PM	87.5	73.8	69.0	63.7
ICD	88.4	53.8	6.5	6.0
CRT	89.1	41.7	11.6	12.5

Abbreviations: CIED = cardiac implantable electronic device, PM = pacemaker, ICD = implantable cardioverter defibrillator, CRT = cardiac resynchronization therapy.

^†^Remaining variance after incremental adjustment for calendar year, age and sex.

^‡^Additionally adjusted for language region, population density, insurance status, and Swiss citizenship.

^§^Additionally adjusted for population health and diabetes mellitus.

^¶^Additionally adjusted for the density of cardiologists.

## Discussion

Our results demonstrate a very high regional variation in PM and ICD implantation rates and a high variation in CRT implantation rates across 25 Swiss HSAs from 2013 to 2016. The largest portion of the variation in implantation rates was explained by demographic, language, socioeconomic factors, and health factors. Whereas these factors explained almost all regional variation in ICD and CRT implantation rates, nearly two thirds of the variation in PM rates remained unexplained.

Not surprisingly, older age was a strong predictor of CIED implantation, most probably as a result of the higher incidence of cardiovascular diseases in the elderly, including sinus node dysfunction [48], heart failure [49, 50], and sudden cardiac death [3]. The decrease in ICD implantation rates after the age of 80 years is consistent with the recommendation to restrict ICD implantations to patients with a minimal life expectancy of at least one year [3–5].

The substantially lower CIED implantation rates in women compared to men (PM– 50%, ICD -78%, CRT -70%) are consistent with the findings of prior studies [51]. Although women show similar improvement in all-cause mortality following PM [51] and ICD [51, 52] implantation and greater survival after the implantation of a CRT [51] compared to men, women were less likely to undergo ICD [24–26, 51, 53, 54], PM [51, 55], and CRT [51, 56, 57] implantations. The lower CIED rates may be at least partially explained by sex-specific differences in the prevalence, clinical presentation, and management of cardiovascular diseases including arrhythmia and heart failure [58, 59]. Overall, women have a lower incidence of sudden cardiac death [60] and coronary artery disease [61], and women with structural heart disease also have a lower incidence and inducibility of ventricular arrhythmias [59]. Another reason for the lower CIED rates in women could be concern for the higher periprocedural complication rate observed in women undergoing ICD [53, 62, 63] and PM [64] implantations. Women with chest pain or an acute coronary syndrome are also less likely to present with typical symptoms, to be referred to a cardiologist [65, 66], to undergo acute revascularization procedures [66, 67], and to receive guideline-recommended drugs than men [66, 68]. Women also suffer more severe heart failure at the time of ICD implantation [54, 62], indicating that ICD procedures are performed at a later disease stage. As women are underrepresented in clinical cardiac treatment trials [69–71] and current guideline recommendations do not address sex-specific differences in their treatment recommendations [1–5], we cannot exclude the possibility that women with cardiac diseases are undertreated compared to men, as described elsewhere [72].

The main determinants of geographic variation were language, socioeconomic factors, and population health. French/Italian speaking language regions had a significantly lower ICD implantation rate (-34%), and somewhat lower PM and CRT implantation rates than Swiss German regions. As French/Italian- and Swiss German speaking regions have a similar cardiovascular morbidity/mortality [29, 73], it appears that cardiologists who practice in the French/Italian speaking areas and who may share a similar training experience are less enthusiastic towards ICD than their Swiss German colleagues. An alternative, albeit unlikely explanation is that patients from French-/Italian speaking regions may have a lower preference for CIED procedures. While our study was not designed to explain differing intervention rates across language regions, we have previously observed lower rates of other preference-sensitive surgical interventions in the French/Italian speaking parts of Switzerland, including vertebroplasty [74, 75], hysterectomy [31], prostatectomy [76], and joint replacement [77] compared to German-speaking regions, indicating that persons living in the Latin speaking parts of the country may be less likely to receive invasive procedures across a broad range of diseases.

Swiss citizenship was associated with a 27% lower CRT rate than foreign citizenship and regions with higher proportion of inhabitants with semiprivate/private health insurance were associated with lower ICD and CRT implantation, demonstrating that socioeconomic factors may have an influential role on procedure rates. Swiss nationals may be more health conscious and have a healthier lifestyle than foreign nationals living in Switzerland [43], resulting in better health and a lower need for CRT. Various immigrant groups living in Switzerland have not only a lower income and health literacy, but also a higher prevalence of cardiovascular risk factors including smoking, overweight, low fruit/vegetable intake, and physical inactivity than non-immigrants [43, 78]. There is an ongoing debate in Switzerland whether semiprivate/private insurance coverage (which results in a higher physician reimbursement) drives procedure rates [79]. Overall, the lower procedure rates among semiprivately/privately insured patients show that a higher insurance level does not fuel overtreatment.

Not unexpectedly, regional population health, expressed as the average number of comorbid conditions, and especially the regional prevalence of diabetes mellitus, were among the strongest determinants of ICD and CRT implantation. In Switzerland, the prevalence of diabetes mellitus varied between 4.4 and 4.8% in 2017 [80] and was substantially lower than in the U.S. (11.1–12.9%) [81]. Diabetes is a strong cardiovascular risk factor [46] and is associated with a 3.8-fold increased mortality within the Swiss population [82].

Factors that may drive procedure rates include financial incentives [83], access to care [28], number of implantation centers [22, 23, 84], and the availability of specialists [85]. While a correlation between the number of cardiologists and implantation rates has been observed in France [85], we did not find an association between the density of cardiologists and CIED rates in Switzerland. Procedure variation may be due to individual physicians’ preferences. Previous studies showed that adherence to guidelines was associated with lower CRT implantation rates [23]. Future studies need to explore potential facilitators and barriers for guideline adherence.

In our study, adjustment for demographics, language, socioeconomic factors, and health explained most of the variance in ICD and CRT device implantation rates. For PM implantation however, the larger portion (63.7%) of the variance remained unexplained by these factors. This finding is in agreement with previous studies that observed a high adherence to guideline recommendations for ICD and CRT [86]. Whereas the decision for ICD and CRT implantations are based on more objective findings (i.e., echocardiography, electrocardiogram, and NYHA functional class [2–5]), PM implantations are often driven by less objective factors (e.g., symptoms and the use of antiarrhythmic drugs that may result in bradycardia) [1, 2]. As patient preferences regarding PM implantation are unlikely to differ across Swiss regions, we hypothesize that regional differences in PM implantation rates may be driven by local physician preferences and practices [27, 39]. Whether CIED implantations are in agreement with guideline recommendations in Switzerland should be further assessed.

Our study has several limitations. First, we used administrative discharge data and cannot exclude the possibility of coding errors. Because CIED data was available for the calendar years 2013–2016 only, determinants for CIED procedures may have changed since then. Second, data about procedure indications and disease severity were not available, precluding any conclusions about the appropriateness of procedure indication. Third, we could not explore other potential determinants of regional variation, such as potential differences in physicians’ and patients’ preferences, training of local physicians, or prevalence of cardiovascular disease on the HSA-level. We can only speculate that local physician and patient preferences may play a role. The underlying reasons for the observed variation warrant further studies as other unmeasured factures may play a role. Fourth, we found associations between CIED rates and several determinants on a regional level, but cannot infer causality on an individual basis. Fifth, the specifics of a country’s health care system may not be necessarily generalizable to other countries. Finally, as many MedStat regions (the smallest geographical unit of our analysis) contain several hospitals, we could not assess the variation of procedure rates between individual hospitals.

In conclusion, we found a very high regional variation in PM and ICD and a high variation for CRT device implantation rates across Swiss HSAs. Differences in demographic characteristics, language/socioeconomic factors, and health explained most of the variance in ICD and CRT device implantation rates. Women had substantially lower implantation rates than men. A large share of the variation in PM procedure rates remained unexplained which might reflect variations in physicians’ preferences and practices.

## Supporting information

S1 Fig Study flow chart. Abbreviations: HSA = hospital service area, PM = pacemaker, ICD = implantable cardioverter defibrillator, CRT = cardiac resynchronization therapy, CIED = cardiac implantable electronic device, ICD10 = International Classification of Diseases, 10^th^ revision, ICD codes X/Y/Z = ICD-10 codes X60–84 (self-harm), Y09–84 (crime related injuries, complications), and Z00–99 (represent reasons for encounters (e.g., vaccination)).
(DOCX)

S1 Table Age- and sex-standardized CIED implantation rates per 100,000 persons by hospital service area. Abbreviations: CIED = cardiac implantable electronic device, PM = pacemaker, ICD = implantable cardioverter defibrillator, CRT = cardiac resynchronization therapy, HSA = hospital service area.
(DOCX)
