# Supplementary figures and images for: Variation in regional implantation patterns of cardiac implantable electronic device in Switzerland
Source: PLoS One. 2022 Feb 16;17(2):e0262959. doi: 10.1371/journal.pone.0262959 (PMC8849475; doi:10.1371/journal.pone.0262959)

**S1 Fig.**


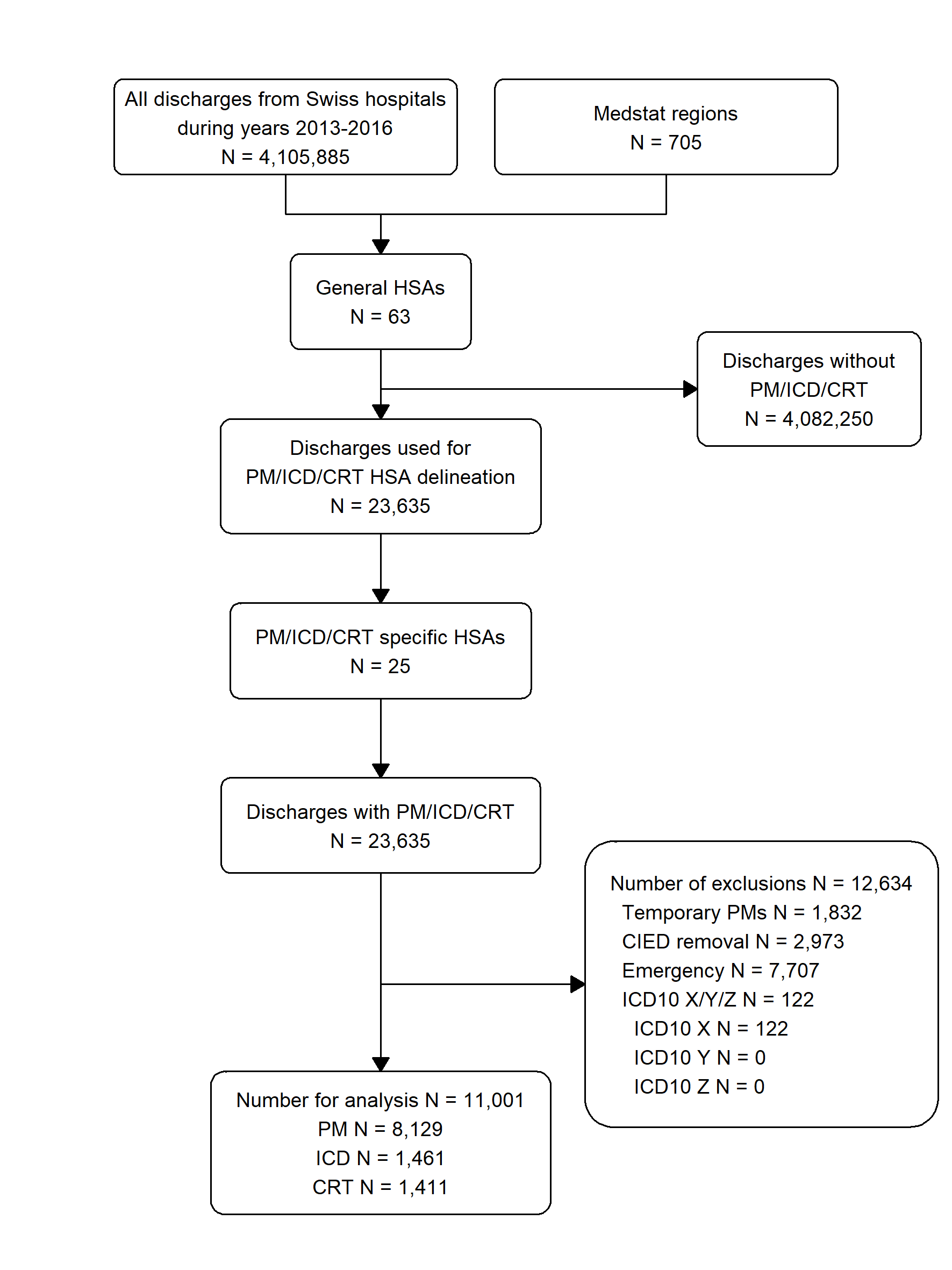

Supplement: S1 Fig — Abbreviations: HSA = hospital service area, PM = pacemaker, ICD = implantable cardioverter defibrillator, CRT = cardiac resynchronization therapy, CIED = cardiac implantable electronic device, ICD10 = International Classification of Diseases, 10th revision, ICD codes X/Y/Z = ICD-10 codes X60–84 (self-harm), Y09–84 (crime related injuries, complications), and Z00–99 (represent reasons for encounters (e.g., vaccination)). (DOCX) [file pone.0262959.s001.docx]
