# Supplementary material for: Variation in regional implantation patterns of cardiac implantable electronic device in Switzerland
Source: PLoS One. 2022 Feb 16;17(2):e0262959. doi: 10.1371/journal.pone.0262959 (PMC8849475; doi:10.1371/journal.pone.0262959)
Supplement: S1 Table — Abbreviations: CIED = cardiac implantable electronic device, PM = pacemaker, ICD = implantable cardioverter defibrillator, CRT = cardiac resynchronization therapy, HSA = hospital service area. (DOCX) [file pone.0262959.s002.docx]

**S1 Table.**

| **PM** | | **ICD** | | **CRT** | |
| --- | --- | --- | --- | --- | --- |
| **HSA** | **Rate** | **HSA** | **Rate** | **HSA** | **Rate** |
| 11 | 57.4 | 8 | 9.2 | 4 | 8.5 |
| 20 | 44.9 | 20 | 8.7 | 3 | 8.0 |
| 9 | 41.4 | 13 | 8.4 | 13 | 7.6 |
| 4 | 39.3 | 15 | 7.8 | 20 | 7.5 |
| 13 | 37.3 | 3 | 6.5 | 21 | 6.6 |
| 8 | 37.1 | 10 | 6.0 | 11 | 6.6 |
| 25 | 36.4 | 11 | 5.9 | 8 | 6.3 |
| 19 | 33.5 | 23 | 5.8 | 17 | 5.9 |
| 3 | 32.6 | 22 | 5.4 | 22 | 5.4 |
| 18 | 30.8 | 9 | 5.2 | 18 | 5.3 |
| 15 | 30.3 | 24 | 5.2 | 19 | 5.3 |
| 10 | 30.2 | 18 | 5.1 | 15 | 5.3 |
| 22 | 30.1 | 21 | 5.0 | 24 | 4.8 |
| 24 | 27.8 | 17 | 5.0 | 7 | 4.8 |
| 17 | 27.0 | 25 | 4.7 | 9 | 4.8 |
| 1 | 26.7 | 19 | 4.5 | 2 | 4.8 |
| 23 | 23.4 | 7 | 4.1 | 23 | 4.7 |
| 16 | 23.4 | 6 | 3.8 | 10 | 4.2 |
| 12 | 22.1 | 4 | 3.7 | 1 | 3.9 |
| 2 | 21.5 | 2 | 3.4 | 6 | 3.7 |
| 7 | 18.8 | 16 | 3.2 | 16 | 3.5 |
| 6 | 16.8 | 14 | 2.6 | 14 | 3.2 |
| 21 | 15.4 | 1 | 2.3 | 5 | 2.6 |
| 5 | 11.4 | 5 | 1.5 | 25 | 2.4 |
| 14 | 8.2 | 12 | 1.3 | 12 | 2.2 |
